# Endostatin and anastellin inhibit distinct aspects of the angiogenic process

**DOI:** 10.1186/1756-9966-27-61

**Published:** 2008-11-04

**Authors:** David M Neskey, Anthony Ambesi, Kevin M Pumiglia, Paula J McKeown-Longo

**Affiliations:** 1 Center for Cell Biology & Cancer Research, Albany Medical College, Albany, New York 12208, USA

## Abstract

**Background:**

Endostatin and anastellin, fragments of collagen type XVIII and fibronectin, respectively, belong to a family of endogenous inhibitors of angiogenesis which inhibit tumor growth and metastasis in a number of mouse models of human cancer. The mechanism of action of these inhibitors is not well understood, but they have great potential usefulness as non-toxic long-term therapy for cancer treatment.

**Methods:**

In this study, we compare the anti-angiogenic properties of endostatin and anastellin using cell proliferation and transwell migration assays.

**Results:**

Anastellin but not endostatin completely inhibited human dermal microvessel endothelial cell proliferation in response to serum stimulation. Both anastellin and endostatin additively inhibited endothelial cell migration in response to VEGF. Anastellin but not endostatin lowered basal levels of active ERK.

**Conclusion:**

These data indicate that anastellin and endostatin exert their anti-angiogenic effects by modulating distinct steps in the angiogenic pathway and suggest that matrix-derived inhibitors of angiogenesis may exhibit higher efficacy when used in combination.

## Background

Angiogenesis, the development of new blood vessels from pre-existing vessels, has been pushed toward the front of cancer research because of its potential therapeutic applications. The angiogenic potential of endothelial cells is determined by a complex balance of positive and negative regulators of growth, migration, invasion and tubulogenesis. Among these regulators are growth factors, such as VEGF, integrin adhesion receptors and extracellular matrix molecules [1,2]. Peptides derived from the extracellular matrix of the tumor microenvironment have been reported to regulate tumor progression and angiogenesis in a variety of mouse models of human cancer and have the potential for developing into promising anti-neoplastic therapies targeting the angiogenic process [3]. Endostatin, a 20 kD terminal fragment of collagen XVIII, is believed to be generated locally in the tumor environment through the action of proteases [4]. Endostatin has exhibited anti-angiogenic properties and anti-tumor activity in a wide variety of human and murine primary and metastatic tumors growing in mice (reviewed in [5]). Although the exact mechanism is unknown, endostatin has been shown to inhibit proliferation and induce apoptosis in some endothelial cell lines [6-9]. Microarray studies have shown that endostatin can regulate up to 12% of all human genes in microvessel cells. Many of these genes are known regulators of angiogenesis [10]. These studies indicate that the effects of anastellin are not linked to one particular signaling pathway, but rather that endostatin impacts a broad network of potentially intersecting pathways important in the angiogenic phenotype. Several receptors have been implicated in mediating the effects of endostatin. These include α5β1 integrins, selectins or cell surface heparan sulfate proteoglycans [11-15].

Anastellin, a 10 kD fragment derived from the first type III repeat of fibronectin (III_1c_), is another anti-angiogenic peptide that inhibits tumor growth and metastasis in vivo [16]. The anti-tumor activity of anastellin has been proposed to result from inhibitory effects on angiogenesis as tumors in anastellin-treated mice exhibit reduced blood vessel density [17]. In vitro, studies using human microvessel endothelial cells show that anastellin inhibits serum dependent cell growth by blocking progression of the cell cycle [18]. The mechanism of action of anastellin is not well understood. It has been reported to bind to α5β1 integrins and proteoglycans [19] and shown to affect the activity of several intracellular signaling molecules [18-21]. Anastellin also binds fibronectin and promotes changes in the organization and assembly of the fibronectin matrix [20,21].

To date, there are no studies which directly compare the effects of these inhibitors on endothelial cell function. We have used human microvessel endothelial cells to compare the effects of anastellin and endostatin on serum-dependent growth and VEGF-dependent cell migration. We find that endostatin and anastellin exhibit distinct effects on microvessel cell proliferation and migration which are likely mediated through differing effects on MAP-Kinase pathways.

## Methods

### Reagents

Unless otherwise indicated, chemical reagents were obtained from Sigma Chemical Co. (St Louis, MO). Recombinant anastellin (III_1C_) was expressed and purified as previously described [21]. Recombinant human endostatin prepared in yeast (Pichia pastoris) was from Molecular Probes (Eugene, OR). Yeast preparations of recombinant human endostatin have been shown to inhibit in vitro angiogenesis and tumor growth [22-24]. Monoclonal antibodies to phospho-ERK (E10) and rabbit polyclonal antibodies to p38 and phospho-p38 (Thr-180/Tyr-182) were obtained from Cell Signaling Tech (Beverly, MA). Rabbit polyclonal antibodies to ERK2 were obtained from Santa Cruz Biotechnology, Inc. (Santa Cruz, CA). Anti-CD146 monoclonal antibody (clone P1H12) was obtained from Chemicon International Inc. (Temecula, CA). Vitrogen-100 was from Cohesion Technologies (Palo Alto, CA).

### Cell culture

Primary adult human dermal microvessel endothelial cells were obtained from VEC Technologies Inc (Rensselaer, NY). Cells were maintained in complete medium [MCDB-131 supplemented with 20% defined fetal bovine serum (D-FBS; HyClone Labs, Logan, UT), 2 mM Glutamax (Gibco), EGM-2MV SingleQuots growth factor cocktail (Cambrex Corp, East Rutherford, NJ), and 10 μg/ml heparin] and cultured on collagen-coated (20 μg/ml Vitrogen-100) tissue culture dishes.

### Cell proliferation assay

Endothelial cells were seeded (500 cells/well) in complete medium (without heparin) onto collagen-coated 24-well plates and allowed to settle for 4 hours. Endostatin and anastellin were added to seeded cells and cells were grown at 37°C in 5% CO_2 _for up to 6 days. At time points, plates were fixed in 3% paraformaldehyde and stored in PBS at 4°C. Endothelial cells were quantified indirectly by ELISA using a mouse anti-endothelial cells (CD146) monoclonal antibody (clone P1H12) as previously described [18].

### Transwell migration assay

Transwell polycarbonate plates with 6.5 mm diameter tissue culture inserts containing a membrane with 8 μm pores were coated with 20 μg/ml of Vitrogen-100 overnight at 37°C in an atmosphere of 5% CO_2_. Following incubation, inserts were washed once with PBS and blocked with 1% BSA/PBS for 1 hour at 37°C. washed and allowed to dry. Cells were suspended in serum free MCDB-131 and seeded (5 × 10^4 ^cells/well) into each insert in the presence of endostatin and/or anastellin. In each outer well, 600 μl of medium with a peptide concentration matching its inner well was added. The plates are then assembled and incubated for 1 hour at 37°C and 10 ng/ml of vascular endothelial growth factor (VEGF) was added to each of the outer wells. After a 4-hour incubation, the plates were rinsed once in PBS, fixed in 3% formaldehyde/PBS for 15 minutes, and rinsed with 0.5% crystal violet. Cells adhering to the top surface of the tissue culture inserts were removed with a cotton tip applicator while cells adhering to the bottom surface of the inserts were rinsed and permeability with 1% Triton-X 100 in PBS for 20 minutes. Subsequently, cells were stained with Hoechst 33258 (1 μg/ml in PBS) for 30 minutes in the dark and then viewed under the fluorescent microscope and the number of cells in 3 random 10× magnification fields was determined.

## Results

### Effects of anastellin and endostatin on endothelial cell proliferation

**Figure 1:**
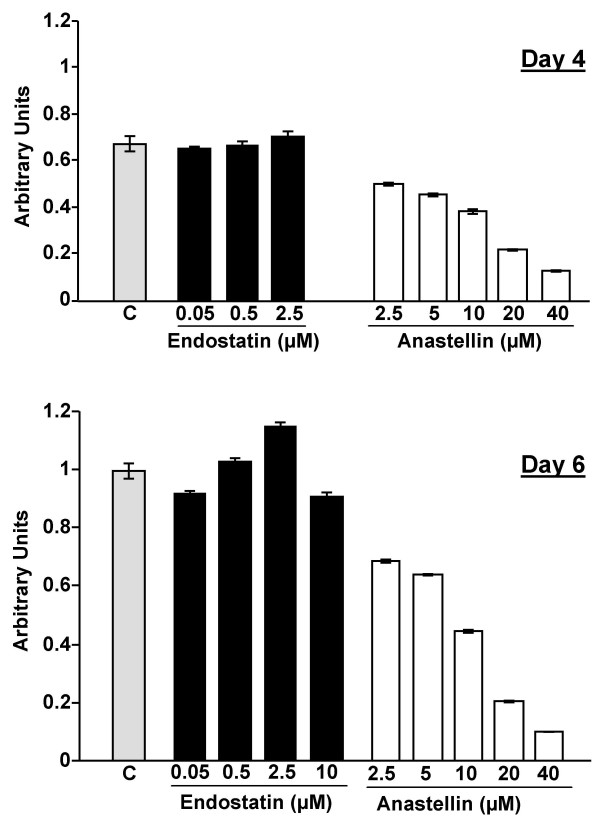
**Effects of anastellin and endostatin on endothelial cell proliferation.** Microvessel cells were seeded (500 cells/well) in the presence of complete medium (day 1). After 4 hours, anastellin or endostatin was added to the medium. At day 4 and ay 6, the number of cells was determined by ELISA. Control wells (C) received no peptide. All wells were normalized to the 6-day control which was set at 1. Error bars represent standard error of the mean of triplicate samples.

Experiments were designed to compare the effect of anastellin and endostatin on microvessel endothelial cell growth. Endothelial cells were seeded in complete medium (20% serum and growth factor cocktail) into collagen-coated wells. Figure 1 shows that the addition of anastellin inhibited serum/growth factor dependent growth in a dose-dependent manner. The inhibitory effects of anastellin on growth were quantified 3 days after seeding (day 4) and again at 5 days after seeding (day 6) (Figure 1). In contrast, endostatin treatment had no effect on microvessel cells which continued to grow at the same rate as control cells. Anastellin treatment did not result in a loss of cells due to disruption of adhesion as cells initially seeded remained attached and spread during the entire 5-day assay (data not shown). These data indicate that in the presence of a complex medium of growth factors, anastellin but not endostatin is a very effective inhibitor of endothelial microvessel cell growth.

### Effect of anastellin and endostatin on endothelial cell migration

**Figure 2:**
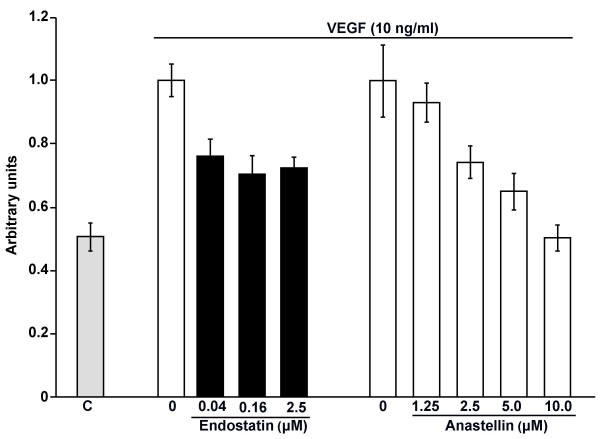
**Effects of anastellin and endostatin on VEGF-dependent endothelial cell migration.** Endothelial cells were seeded on collagen-coated transwell tissue culture inserts in the presence of either anastellin or endostatin, which was added to both upper and lower chambers. Control wells (C or 0) received no peptide. Positive control wells (0) were set at 1 and represent VEGF migration. After 1 hour, the 10 ng/ml VEGF was added to the lower chamber as chemoattractant. After 4 hours, the plates were fixed, stained and cells in three 10× fields counted in each of 3 membranes. The bars represent standard error of the mean from 3 separate experiments (n = 27).

To evaluate the effects anastellin and endostatin on endothelial cell motility, microvessel cells were seeded onto collagen-coated filters in modified Boyden chambers. As shown in Figure 2, the addition of VEGF to the bottom chamber as a chemoattractant resulted in a two-fold increase in the number of cells migrating to the bottom of the filter. Addition of anastellin caused a dose-dependent decrease in VEGF-stimulated cell migration. Complete inhibition of VEGF-stimulated migration was seen at 10 μM anastellin. Endostatin also inhibited VEGF-induced migration, but the effects were only partial. Endostatin inhibited VEGF-induced migration by 40–50% over the dose range tested. This result is consistent with previous studies where endostatin was shown to inhibit VEGF stimulated migration by 60–70% [25,26].

**Figure 3:**
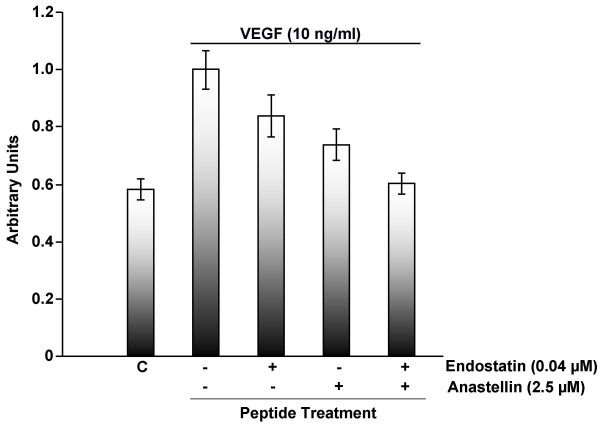
**The effects of anastellin and endostatin on endothelial cell migration are additive.** Endothelial cells were seeded on collagen-coated transwells in the presence of endostatin or anastellin either individually or in combination. Peptides were added to both chambers at the time of seeding. After 1 hour, 10 ng/ml VEGF was added to the lower chamber. Control wells (C) received no peptides and no VEGF and represent baseline migration. Positive control wells (+) were set at 1 and represent VEGF dependent migration. After 4 hours, plates were fixed, stained and the number of cells migrating to the underside of the filter were counted in three 10× fields in each of triplicate wells. Bars represent standard error of the mean from data obtained from 7 separate experiments, n = 63.

Experiments were done to address whether the effects of anastellin and endostatin on cell migration were additive. Based on the results shown in Figure 2, doses of each peptide were selected which would give partial inhibition of migration. As shown in Figure 3, 0.04 μM endostatin inhibited migration by approximately 35%, 2.5 μM anastellin inhibited migration by approximately 60%. When added together, there was greater than a 95% inhibition of migration. These data indicate that the effect of these inhibitors on migration are additive and suggest that they exert independent effects on cell migration.

### Effect of endostatin and/or anastellin on basal levels of MAP kinase activity

**Figure 4:**
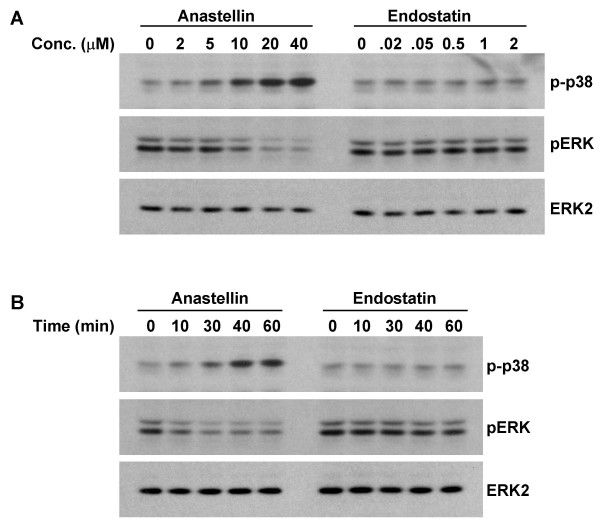
**Effect of anastellin and endostatin on ERK and p38 MAP kinase activity.** Micro-vessel cell monolayers were treated with the indicated doses of either anastellin or endostatin for 1 hour (A) or treated with 20 μM anastellin or 0.5 μM endostatin for the indicated time (B). Cell layers were washed twice with ice-cold PBS containing 1 mM Na_3_VO_4_, solubilized in gel sample buffer. Cell lysates were prepared and analyzed by Western blot for phospho-ERK and phospho-p38 MAP kinase. Blots were then stripped and reprobed for either total ERK as a loading control. Additional controls indicated no change in the levels of p38 (data not shown).

As ERK and p38 MAP kinase have been reported to regulate growth and migration of endothelial cells in response to growth factors including VEGF [27-29], we compared anastellin and endostatin for their effects on the basal activities of these MAP kinases in microvessel cells. Cells were incubated with either anastellin or endostatin for 1 hour. The doses used were those shown to inhibit either proliferation (Figure 1) or migration (Figure 2). Following treatment, cell lysates were analyzed for active ERK (pERK) or p38 MAP kinase (p-p38) by Western blot. Figure 4A shows that increasing doses of anastellin caused a marked increase in the activation of p38 and a nearly complete loss of active ERK. The effects of anastellin on the MAP kinase activities were dose-dependent between 5–20 μM and correlated well with the amounts of anastellin required to inhibit cell proliferation and migration (Figures 1 and 2). In contrast, endostatin had no effect on the levels of active p38 or ERK when used at doses shown to cause an inhibition of migration (Figure 2). Figure 4B shows that the effects of anastellin on MAP kinase activity occurred within minutes. Maximal activation of p38 was seen between 30–40 minutes, while inhibition of ERK was seen by 10 minutes. Consistent with the results shown in Figure 4A, endostatin and anastellin exert differential effects on the activity of ERK and p38 MAP kinases and suggest that these peptides inhibit angiogenesis by modulating distinct signaling pathways in microvessels cells.

## Discussion

The present study shows that anastellin, but not endostatin, is an effective inhibitor of microvessel cell growth in response to growth factor supplemented serum. These findings differ from earlier studies showing that endostatin could inhibit bFGF- or VEGF-stimulated endothelial cell growth [6,28,30-33] or in vivo angiogenesis in response to VEGF [34]. The discrepancy between these earlier results and the current data may be due to differences in the assay system (serum-dependent growth) or in the cell type (human microvessel endothelial cells). In agreement with this, other studies have shown that the effects of endostatin on endothelial cells can be quite varied depending on the endothelial cell source [35]. Although microarray studies have shown that endostatin targets a large number of genes [36], our studies suggest that anastellin effects a broader base of targets to include those important in proliferation of endothelial cells. Taken together, these data suggest that endostatin may selectively inhibit signaling through individual growth factor receptors but it may be less effective in inhibiting growth in response to a more complex mixture of growth stimulatory signals. Anastellin and endostatin have both been reported to bind to α5β1 and heparan sulfate proteoglycans [12,19,37]. This would suggest that these two peptides might exert similar effects on cell behavior. However, in this study we have found that these two peptides exhibit distinct effects on both cell behavior and MAP kinase pathways. Consistent with this observation, we have found more recently that the effects of anastellin on p38 are independent of β1 integrins [38].

The effects of anastellin and endostatin on cell migration indicate that combined regimens of matrix-derived peptides and provide additive levels of inhibition. Other angiogenesis inhibitors derived from matrix molecules regulate effects on endothelial cells through a variety of mechanisms. Tumstatin, a fragment of Type IV collagen, inhibits endothelial cell proliferation and induces apoptosis through the mTOR pathway but has no effect on cell migration [39]. Canstatin, another fragment of the Type IV collagen, has also been shown to inhibit serum-dependent cell proliferation and induce apoptosis. Unlike anastellin, canstatin's inhibition of cell proliferation was not associated with changes in ERK activity but were dependent upon apoptotic signaling events transduced through membrane death receptors [40,41]. In contrast to other Type IV collagen fragments, arrestin which is derived from the α1 chain of type IV collagen does not induce apoptosis but inhibits endothelial cell proliferation and migration and their associated signaling pathways including ERK1/2, FAK, and p38 MAPK [42]. As each of these matrix-derived peptides activate distinct anti-angiogenic pathways, it is probable that combinations of matrix-derived peptides would result in synergistic inhibition of not only cell migration but also of neovascularization in general [43,44]. In addition, these peptides may augment the anti-tumor effects of more traditional chemotherapeutic agents or oncolytic viruses [45-49].

The basis for endostatin's or anastellin's inhibitory effects on tumor growth and metastasis in vivo may extend beyond direct effects on the tumor vasculature to more widespread effects on the tumor microenvironment. Endostatin has been shown to exhibit direct effects on tumors. Endostatin treated mice undergoing carcinogen-induced skin tumors exhibit less aggressive more differentiated tumors, suggesting that endostatin regulates terminal differentiation of keratinocytes [50]. When given in combination with angiostatin during the early states of prostate cancer in the TRAMP mouse, endostatin arrested the progression of moderately differentiated carcinoma to poorly differentiated carcinoma [51]. Anastellin has also been shown to activate signaling pathways in dermal fibroblasts, suggesting that anastellin may elicit biologic effects on stromal cells present within the tumor [20,21]. The extravascular effects of matrix-derived inhibitors of angiogenesis within the tumor microenvironment represent an important area of future investigation.

## Conclusion

Our data indicate that the matrix-derived inhibitors of angiogenesis, endostatin and anastellin, exhibit effects on endothelial microvessel cell proliferation and migration which are associated with differing effects of MAP kinase activity. These findings suggest that combinatorial anti-angiogenic therapies may provide novel treatments for the management of cancer as a chronic disease.

## Competing interests

The authors declare that they have no competing interests.

## Authors' contributions

DMN and AA performed experiments and provided statistical analysis. KMP provided technical assistance, experimental design and data interpretation. PJM-L provided conceptual oversight on the project including manuscript design and interpretation of experiments as well as responsibility for final draft of the manuscript.
